# Human placental mesenchymal stromal cell‐derived exosome‐enriched extracellular vesicles for chronic cutaneous graft‐versus‐host disease: A case report

**DOI:** 10.1111/jcmm.17114

**Published:** 2021-12-06

**Authors:** Amir Hossein Norooznezhad, Reza Yarani, Mehrdad Payandeh, Zohreh Hoseinkhani, Sarah Kiani, Elham Taghizadeh, Avnesh S. Thakor, Kamran Mansouri

**Affiliations:** ^1^ Medical Biology Research Center Health Technology Institute Kermanshah University of Medical Sciences Kermanshah Iran; ^2^ Translational Type 1 Diabetes Research Department of Clinical Research Steno Diabetes Center Copenhagen Gentofte Denmark; ^3^ Department of Radiology Interventional Regenerative Medicine and Imaging Laboratory Stanford University School of Medicine Palo Alto California USA; ^4^ Bone Marrow Transplantation Department School of Medicine Kermanshah University of Medical Sciences Kermanshah Iran; ^5^ Department of Dermatology School of Medicine Razi Hospital Tehran University of Medical Sciences Tehran Iran

**Keywords:** cutaneous GVHD, exosome, graft‐versus‐host disease, human mesenchymal stromal cell

## 1 BACKGROUND

Allogeneic peripheral blood stem cells transplantation (PBSCT) is now among the treatments for different haematological diseases and malignancies such as acute myeloid leukaemia (AML).^1^, ^2^ Although this treatment is very advanced, graft‐versus‐host disease (GVHD) is still the most common and severe side effect.^3^, ^4^ Considering the time course, GVHD can be divided into two forms: acute and chronic.^4^, ^5^ The cumulative incidence of chronic GVHD (cGVHD) during the first year of transplantation is as high as 43.8 ± 10% among recipients of allogeneic marrow transplantation.^6^ Moreover, studies have shown a 57% cumulative 5‐year incidence of cGVHD in patients who have undergone haematopoietic stem cell transplants from their siblings.^7^ Clinically, cGVHD involves mainly skin, lung, eye, liver and musculoskeletal system,^4^, ^8^ in which the cutaneous type is the most prevalent and earliest exhibiting type. According to the studies, the cutaneous cGVHD is associated with an inflammatory state in which anti‐inflammatory agents are the most important treatment option.^5^

Human mesenchymal stromal cells (hMSC) have been used to treat many inflammatory diseases/disorders in the clinic. A recent systematic review and meta‐analysis showed that treatment with these cells is not associated with any severe or notable side effects.^9^ Exosomes are natural extracellular vesicles released by different cell types and contain proteins, lipids and RNA. These vesicles have been known to participate in intercellular interactions and communications. Depending on the origin and microenvironment of exosomes, they could play different roles, of which immune modulation is one of the most important ones.^10^

Comparing to other sources such as bone marrow or adipose tissue, the placenta is a richer source of stem cells. Since in pregnancy, there is a ‘tolerated allograft’ in which the placenta is the immunoregulatory organ in this process, this tissue might be a better source in the allogeneic stem cell source. This issue has been shown in the xenotransplantation of this tissue, which resulted in low immunogenicity in immunocompetent animals. Furthermore, placenta cells lack MCH class II antigens, which are responsible for allograft rejection. These and many other reasons make the placenta an immune privileged tissue to isolate mesenchymal stem/stromal cells.^11^

This study aims to report a case of cutaneous cGVHD who received human placental mesenchymal stromal cells (hPMSC)‐derived exosome‐enriched extracellular vesicles (EVs) as a treatment option.

## 2 METHODS AND PATIENT

### 2.1 Cell isolation

hPMSCs derived from human placenta (single donor) tissue were isolated and identified by the method has been described by Pelekanos et al.^12^ Also, the donor's blood sample was negative for viral infections including mycoplasma, cytomegalovirus (CMV), hepatitis B virus (HBV), hepatitis C virus (HCV) and human immunodeficiency virus (HIV) evaluated by polymerase chain reaction (PCR). The cells exhibited surface expression of mesenchymal markers (CD73, CD 105, CD90 and CD 44; Figure S1) and were negative for haematopoietic markers (CD45, CD 34 and HLA‐DR) identified as hPMSCs. Also, the cells at passages 2–3 were assessed for osteogenic, adipogenic and chondrogenic differentiation potentials.

### 2.2 Exosome purification and characterization

**FIGURE 1:**
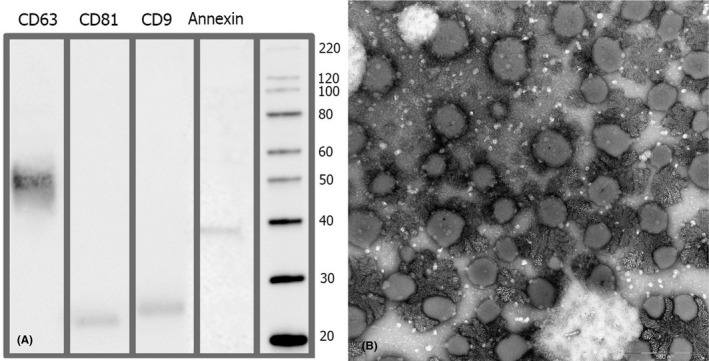
(A) Western blot analysis of purified extracellular vesicles. The lines are showing CD63, CD81, CD9 and annexin as markers for extracellular vesicles form the human placental mesenchymal stem cells. Numbers. (B) Transmission electron microscopy of purified exosomes‐enriched extracellular vesicles

hPMSCs passages 3–6 were maintained in Dulbecco's minimal essential medium (DMEM) supplemented with 10% Human AB serum (mycoplasma, CMV, HBV, HCV and HIV tests performed by PCR), 100 U/ml penicillin and 100 µg/ml streptomycin and incubated at 37°C in 5% CO_2_. Cells were grown to 70%–80% confluency, washed three times with PBS and incubated for 48 h in serum‐free media (low‐glucose DMEM/F12). In the presence of a cellular density of 80%, the hPMSCs‐conditioned media were harvested every 48 h. To sterilize the supernatants and remove larger EVs, the conditioned media were passed through 0.22 mm filter membranes and centrifuged at 700 *g* for 10 min at 4°C to remove cell debris. Another round of centrifugation was performed at 9000 *g* at 4°C for 30 min, and the supernatant was collected again. Exosomes were isolated by ExoEasy Maxi kit (76064; Qiagen) and resuspended in phosphate‐buffered saline (PBS). Residual polyethylene glycol and soluble proteins that might have been coprecipitated were removed, pellets were washed with 0.9% NaCl and reprecipitated at 100,000 *g* at 4°C for 2 h. The obtained pellets were then dissolved in 0.9% NaCl to a final concentration of 1 unit EVs per 1 ml and stored as 1 ml aliquots at −80 C until usage. The characterization of exosomes was achieved by measuring expression of exosome‐specific markers CD81, CD9, nex1 and CD63 by Western blot analysis (Figure 1A ) and particle size by NanoSight analysis (platforms SLM 20). The concentration of exosome‐enriched EVs was determined by analysing protein concentration using the Bradford protein quantitation assay kit (Zist Tolid Razi) with BSA as a standard.

The yield of an EV fraction prepared from supernatants of 4–6 × 10^7^ hPMSCs that had been conditioned for 48 h was defined as 1 unit. The EV content of the four processed hPMSCs supernatants was determined by NanoSight. The average particle sizes calculated varied for the different preparations between 110 and 130 nm. The protein and particle content of the three independent hPMSCs supernatant fractions were all in the same ranges (0.5–0.8 mg/unit; 1.9 × 10^11^–2.6 × 10^11^ particles/unit). Western blot analyses determined the exosomal marker proteins CD81, CD9, nex1 and CD63 in all fractions. Together with the vesicular appearances of obtained particles within transmission electron microscopy (Figure 1B), these findings indicated that the obtained samples were highly enriched in exosomes. Also, endotoxin‐sensitive gel‐clot LAL test (0.03 EU/ml) and viral tests, including mycoplasma, CMV, HBV, HCV, HIV tests and 14‐day sterility (Gram stain negative), were used as product release criteria. Exosome solution was infused within 3 h of preparation for infusion.

## 3 CASE PRESENTATION

The patient was a 39‐year‐old Caucasian male diagnosed with AML type M4 5 years ago (April 2016). Following routine treatments and after reaching complete remission, he underwent PBSCT (one session) from an identical donor (brother). After the transplantation, he presented with acute gastrointestinal (GI) GVHD on a prophylaxis immune suppression regime, with his symptoms and signs brought under control through increasing corticosteroid and cyclosporin dosages. After a year, the cutaneous cGVHD started, which did not respond to extracorporeal photopheresis (12 sessions), tacrolimus, imatinib and high‐dose corticosteroids. Also, during the last 18 months, the patient was receiving 5 mg per day of prednisolone and 50 mg and 25 mg per odds and even days (respectively) of cyclosporine for 18 months.

**FIGURE 2:**
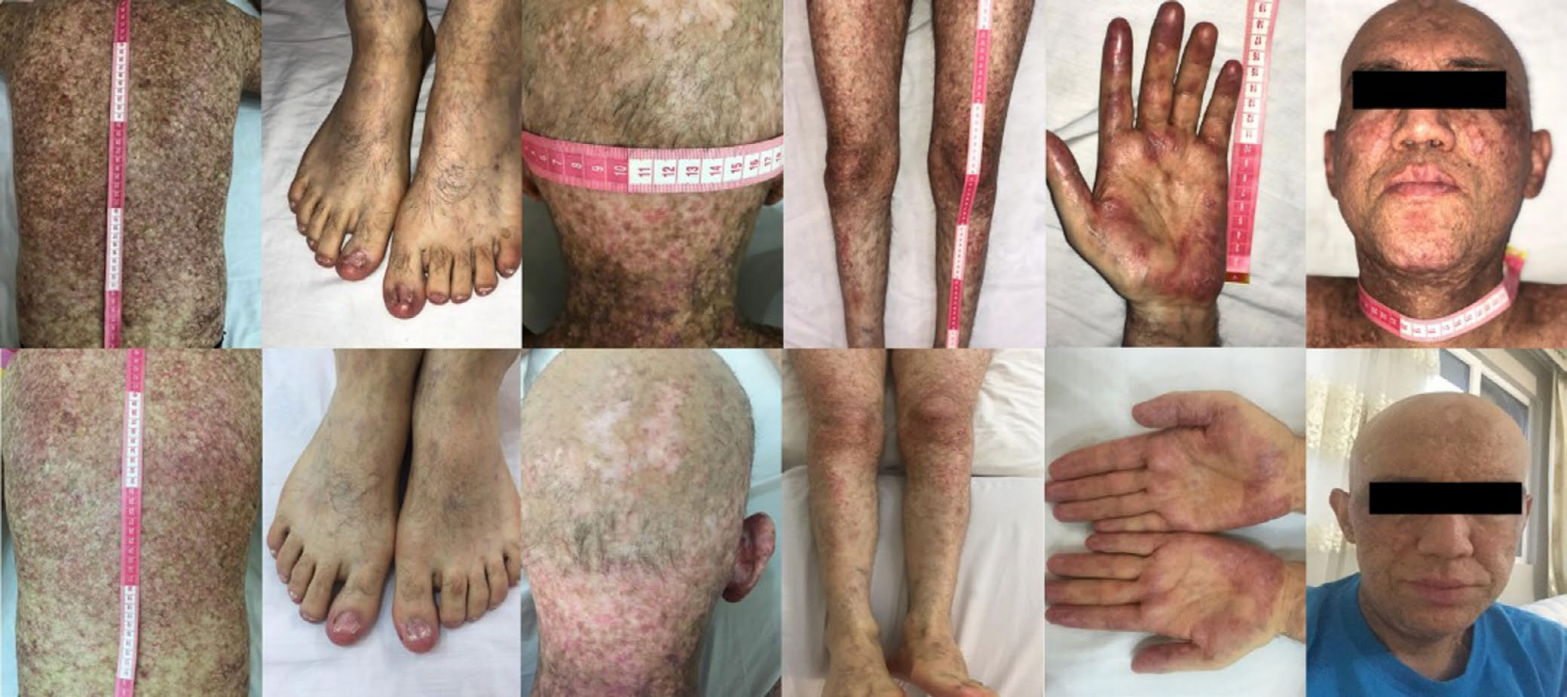
Cutaneous chronic graft‐versus‐host disease in the patient. Upper and lower pictures represent before and after the treatment (four treatments of extracellular vesicles therapy)

At his clinic visit, reticulated pink to violet papules and plaques admixed with post‐inflammatory hyperpigmentation were noted on his face and other areas. Moreover, vitiligo/amelanic patches with scarring alopecia were detected on his scalp. Psoriasiform and eczematous‐like lesions were also noted on his lower limbs, with extensive involvement of the back and trunk in the form of poikiloderma was observed. Nail involvement was also detected in different forms, including ridging, thinning, splitting, onycholysis and anonychia. The palmar and ventral sides of his forearm were involved in the form of dyspigmentation, hair loss and multiple erosion and ulceration. Furthermore, long‐standing fibrosis seemed to have caused skin ulceration, particularly in legs and frictional surfaces (Figure 2). Also, the cGVHD of the patient decreased from II to I (mild). Laboratory evaluations noted a monocyte ratio (18%: 1229 cells/mm^3^) accompanied with normal lymphocyte and neutrophil count, increased haemoglobin and erythrocytes sedimentation rate (ESR), aspartate transaminase (AST), alkaline transaminase (ALT), alkaline phosphatase (ALP) and triglyceride (TG) (see Table 1).

**TABLE 1:** Laboratory variables before and 4 weeks after the last session of intervention

Variable	Before treatment	After the fourth intervention
White blood cells/mm^3^	6830	7800
Neutrophils/mm^3^	2049	3666
Lymphocytes/mm^3^	3346	3588
Monocytes/mm^3^	1229	390
Eosinophils/mm^3^	204	2
Haemoglobin gr/dl	17.7	17.3
Haematocrit (%)	51.1	48.3
Platelet ×10^3^/mm^3^	225	228
C‐reactive protein	1+	Negative
Erythrocyte sedimentation rate (mm/h)	23	10
Creatinine mg/dl	0.8	0.9
Aspartate transaminase IU/L	59	57
Alkaline transaminase IU/L	50	59
Alkaline phosphatase U/L	333	365
Total bilirubin	0.8	0.8
Direct bilirubin	0.4	0.4
Lactate dehydrogenase U/ml	482	461

The exosomes‐enriched EVs were isolated from placental‐derived human mesenchymal stromal cells as has been described, and the patient received four treatments at a weekly interval (June 2021; 5 years and 2 months after the transplantation). In each session, 0.5–0.8 mg (1.9–2.6 × 10^11^ particles) of exosome‐enriched EVs were administrated in 50 ml saline (0.9%) through the right cubital vein access. The patient well tolerated the treatment, and no side‐effect was observed following the intervention. Also, no infection was noted during the treatment and follow‐up period. The changes began after the third injection, and on the 15th day following the last intervention, when he began to feel significant changes in his condition, the patient was evaluated more closely. As shown in Figure 2 (15 days following the last injection), his skin has become less hyperpigmented. Also, the frequency and severeness of the mentioned ulcers, wounds and keratotic and atrophic lesions decreased, and healing of the wounds was observed. Moreover, the stiffening and dryness of the skin were significantly improved following the treatment. Also, the patient was very stratified following the intervention. The changes in the laboratory variables have been gathered in Table 1. The patient was followed for 5 months, and the mentioned changes remained sustained.

## 4 DISCUSSION

This study aimed to evaluate the potential of treatment with hPMSC‐derived exosomes‐enriched EVs on a previously known case of cutaneous cGVHD. It was shown that the mentioned treatment could decrease the signs and symptoms caused by cutaneous cGVHD, specifically hyperpigmentations and ulcers caused by skin dryness. Also, the cutaneous inflammation decreased significantly and was more evident than other manifestations due to the anti‐inflammatory potential of the treatment.

As shown in Table 1, monocytes have been decreased from 18% to 5%, which is clinically significant. As mentioned, the patient was resistant to the many treatment options, including the corticosteroids. It has been shown that donor monocytes could be involved in the pathogenesis of GVHD. In patients diagnosed with GVHD, it has been shown that the intermediate CD14^++^ CD16^+^ monocytes could promote the induction of a subset of Th17 glucocorticoid resistance cells.^13^ Thus, it seems that our intervention was able to reduce this effect in our patient who did not respond to corticosteroid therapy.

Human mesenchymal stromal cells have been used to treat GVHD in patients undergoing haematopoietic stem cell transplantation. Bonig et al. have used these cells to treat cases with acute refractory GVHD (R‐aGVHD). In their study, 92 patients diagnosed with R‐aGVHD were enrolled in which two‐thirds were therapy‐resistant patients, and one‐third were only steroid refractory cases. The authors declared that no toxicity was observed following the intervention (median of three doses). It was shown that the overall response rate was 82% in the first evaluation.^14^

The only case of GVHD treatment by exosome therapy has been explained by Kordelas et al. They have reported a case of GVHD following PBSCT (HLA‐identical female donor) due to the myelodysplastic syndrome, which led to mucosal GVHD. Also, due to the secondary AML, she received allogenic PBSCT from an HLA‐identical male donor, leading to hyperacute cutaneous GVHD (grade IV). The patient finally expressed severe intestinal and skin GVHD and underwent bone marrow‐derived MSC exosome therapy. The patient showed a notable response to the treatment. The cutaneous and mucosal GVHD showed initial changes from week 2, which lasted 4 months (followed by a decrease in the steroid dose). Unfortunately, their patient expired 6 months later due to pneumonia.^15^

To our knowledge, we are reporting the second case of exosome therapy for GVHD in a cutaneous cGVHD patient, which showed clinically acceptable results for both the team and the patient. The results remained stable for 4 months with no relapse. This study only investigated the hPMSC exosome therapy due to the lack of resources. The authors suggest investigating differences among other sources of MSCs such as bone marrow for cGVHD treatment. Also, changes in the environment of the MSCs could be considered as other variables in future studies.

## 5 CONCLUSION

Although many studies have used mesenchymal stem cells in clinics, data on using MSCs exosome therapy for GVHD are extremely limited. The experience of our team with hPMSC exosome‐enriched EVs therapy on the described cutaneous cGVHD patient was clinically successful, making it a potential treatment for this pathology. However, further complementary studies and trials are strongly suggested.

## CONFLICT OF INTEREST

The authors declare no actual or potential conflict of interest related to this study.

## AUTHOR CONTRIBUTIONS

**Amir Hossein Norooznezhad:** Conceptualization (equal); Investigation (equal); Writing – original draft (equal); Writing – review & editing (equal). **Reza Yarani:** Investigation (equal); Writing – original draft (equal); Writing – review & editing (equal). Mehradad Payandeh: Investigation (equal); Writing – original draft (equal); Writing – review & editing (equal). **Zohreh Hosseinkhani:** Investigation (equal); Writing – original draft (equal); Writing – review & editing (equal). **Sarah Kiani:** Investigation (equal); Writing – original draft (equal); Writing – review & editing (equal). **Elham Taghizadeh:** Investigation (equal); Writing – original draft (equal); Writing – review & editing (equal). **Avnesh Thakor:** Investigation (equal); Writing – original draft (equal); Writing – review & editing (equal). **Kamran Mansouri:** Conceptualization (equal); Methodology (equal); Supervision (equal); Writing – original draft (equal); Writing – review & editing (equal).

## CONSENT

The patient signed a consent form freely and agreed for us to publish his records and receive the interventions after explaining the aims and methods of study according to his level of education and knowledge.

## Supporting information

Fig S1

## Data Availability

Data would be available through online request to the corresponding author Dr. Kamran Mansouri.
