# Supplementary figures and images for: Human placental mesenchymal stromal cell‐derived exosome‐enriched extracellular vesicles for chronic cutaneous graft‐versus‐host disease: A case report
Source: J Cell Mol Med. 2021 Dec 6;26(2):588–92. doi: 10.1111/jcmm.17114 (PMC8743661; doi:10.1111/jcmm.17114)

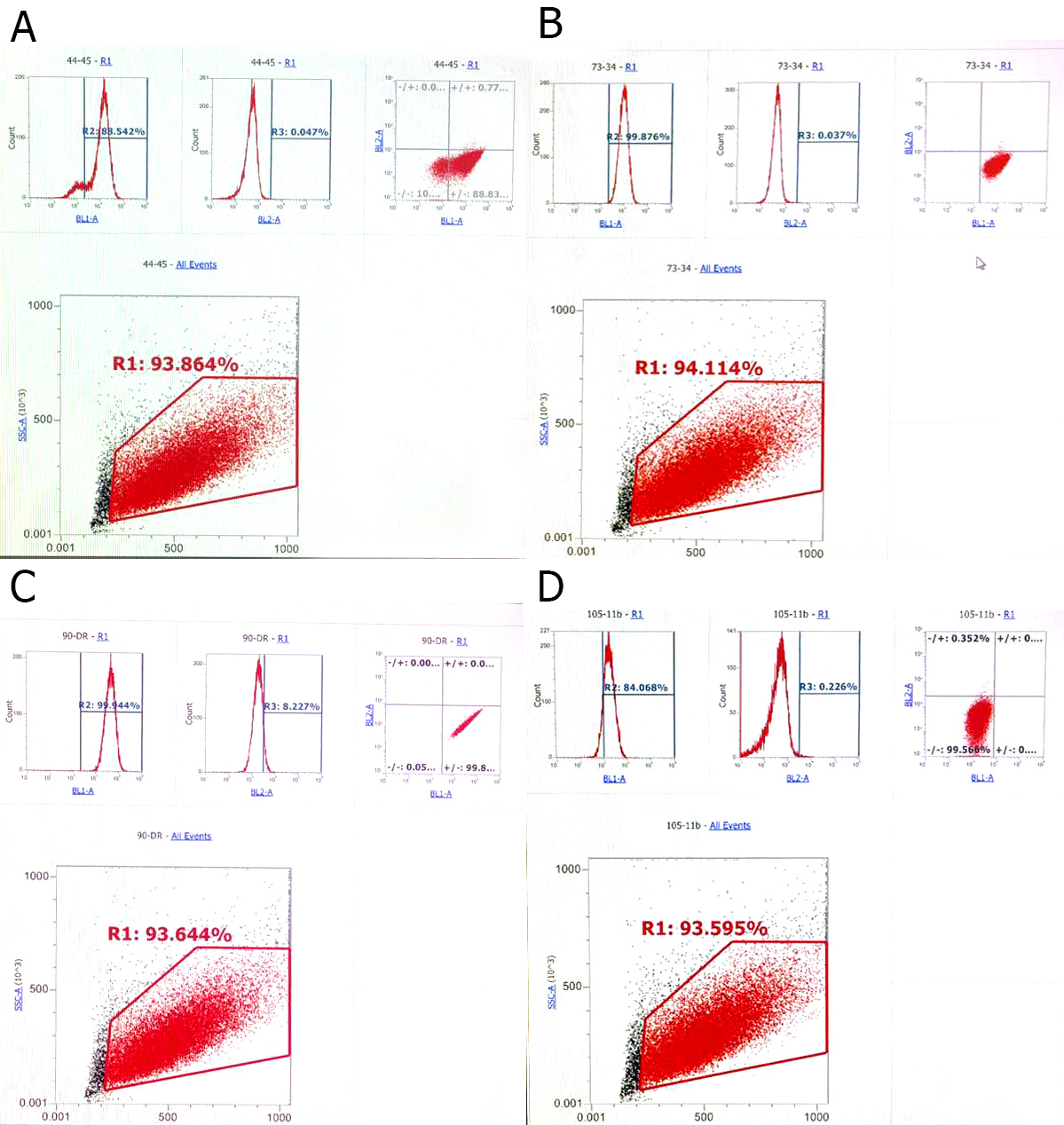


Figure S1. Flow cytometry of the human placental mesenchymal stromal cells. A: CD 44. B: CD 73. C: CD 90.D: CD 105.

Supplement: Supplementary file 1 — Fig S1 [file JCMM-26-588-s001.docx]
